# Conus stabilization for pulmonary valve reconstruction after transannular patch

**DOI:** 10.1016/j.xjtc.2025.06.014

**Published:** 2025-06-26

**Authors:** Igor E. Konstantinov, Carolina Rodrigues, Damien Wu, Segrei I. Konstantinov

**Affiliations:** a Department of Cardiothoracic Surgery, Royal Children's Hospital, Melbourne, Victoria, Australia; b Department of Paediatrics, University of Melbourne, Melbourne, Victoria, Australia; c Heart Research Group, Murdoch Children's Research Institute, Melbourne, Victoria, Australia; d Melbourne Centre for Cardiovascular Genomics and Regenerative Medicine, Melbourne, Victoria, Australia

**Figure:**
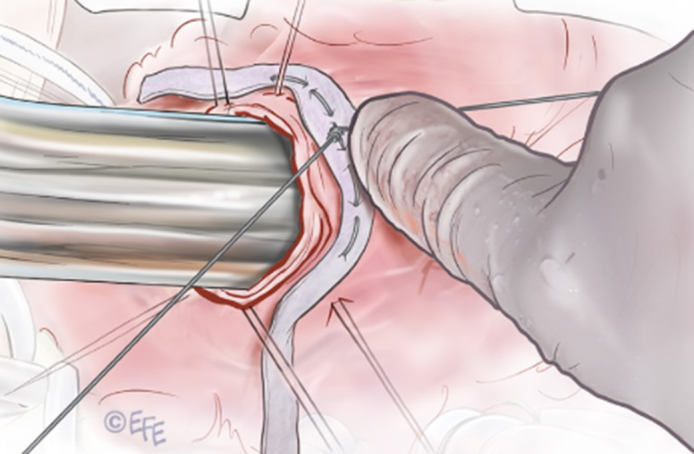
Stabilization of the subvalvular muscular conus around Hegar dilator.

**Central Message**

Subvalvular muscular conus can be stabilized at the desired diameter.

Pulmonary valve replacement (PVR) is 5 times more common than any other valve replacement in pediatric cardiac surgical practice.^1^ There is a growing population of adolescents and adults with repaired congenital heart disease with severe pulmonary insufficiency and dilatation of the right ventricle (RV) and its muscular conus component.^2^ Traditionally, these patients undergo PVR with either homograft or prostheses. It has been notoriously difficult to repair pulmonary valves in this subgroup of patients due to persistent dilatation of the muscular conus. Herein we describe a simple technique of conal stabilization that reduces the subvalvular muscular conus to a desired diameter.

## Case Report

A 15-year-old adolescent (weight, 68 kg; height, 161 cm; body surface area, 1.72 m^2^) had tetralogy of Fallot (TOF), with bicuspid pulmonary valve, that was repaired at age 6 months with a transannular patch, and now presented with exercise intolerance, severe pulmonary regurgitation, and progressive RV dilatation. The parents provided written consent for publication of the data and the study was approved by the hospital ethics committee (HREC/21/QCHQ/80891 on November 11, 2021).

The preoperative echocardiogram (Video 1) demonstrated dilated RV with reduced systolic function and severe pulmonary regurgitation. Cardiac magnetic resonance imaging demonstrated normal left ventricular volumes and systolic function, RV end-diastolic volume index of 115 mL/m^2^ (RV to left ventricle volume ratio ∼ 2:1) with preserved systolic function, unobstructed RV outflow tract (RVOT), and pulmonary regurgitation fraction of 42%.

## Operative Technique

**Figure 1:**
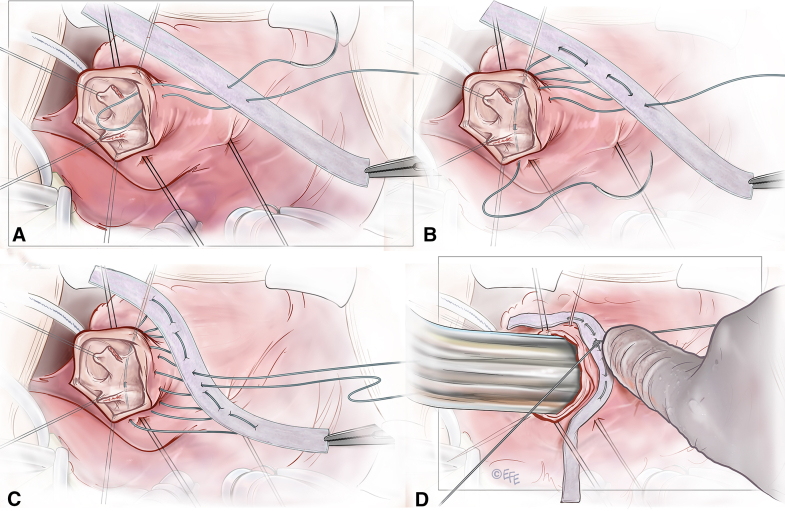
Technical details of the stabilization of the conus and reconstruction of the pulmonary valve. The GoreTex suture (W. L. Gore & Associates) (CV-0) is placed through the polytetrafluoroethylene strip (A), passed immediately below the pulmonary valve (B), and then comes out and passes via the polytetrafluoroethylene strip again (C). The suture is tightened around Hegar dilator to stabilize the subvalvular muscular conus at the desired diameter (D).

Surgery was performed on the beating heart. The main pulmonary artery (PA) was opened and both cusps were examined (Video 1). The largest Hegar dilator size (27 mm in diameter) went via the subvalvular area without any restriction. A circular suture of GoreTex CV-0 (W.L. Gore & Associates) was placed in the subvalvular muscular conus and external teflon strip reinforcement. The suture was tightened around the Hegar dilator and stabilized at the desired diameter (Figure 1). Both cusps of the PV were detached from the annulus at the nadir of each cusp. Each cusp was reconstructed with a Cardiocel patch (Admedus, Ltd), creating a neocommissure anteriorly. The newly created commissure was suspended and sutured to the Cardiocel patch used to reconstruct the main PA anteriorly. Postoperative echocardiogram demonstrated mild regurgitation and no significant gradient in the RVOT (Video 1), which remained unchanged at 6 months.

## Discussion

In our recent retrospective study of 960 patients with repaired TOF, 8.6% underwent surgical PVR with the median time interval from initial TOF repair to PVR being 14 years.^3^ The prostheses used for the first implantation were pulmonary homograft (n = 43), bovine jugular valved conduit (n = 27), Medtronic Freestyle bioprosthesis (n = 10), and others (n = 3).^3^ We previously demonstrated in a retrospective review of 586 patients who underwent an RV-PA conduit, that pulmonary homografts perform better than aortic homografts or bovine jugular vein grafts, in patients younger than age 20 years and conduits larger than 15 mm.^4^ Freedom from reintervention at 5 and 10 years was 92% and 80%, respectively, for pulmonary homografts, 74% and 37% for bovine jugular veins, and 75% and 47% for aortic homografts.^4^

Several options for PVR have been described, including Ozaki-type PVR^5^ and percutaneously implanted valves. Feasibility of percutaneous pulmonary valve implantation depends on stability of the pulmonary valve annulus. Thus, severely enlarged subvalvular muscular conus makes the feasibility of percutaneous valve insertion questionable. This was emphasized in a multicenter study with 229 attempted Melody valve (Medtronic) implants, where the most frequent reason for failure of implantation was an RVOT diameter exceeding Melody valve maximum diameter.^E1^ No implants were performed in patients with RVOT dimensions >22 × 23 mm, measured on cross-sectional imaging.^E1^ The Melody valve has a rated outer diameter of 24 mm, with capacity for overdilation to reach a maximum diameter of 25 mm.^E2^ The Sapien valves (Edwards Lifesciences) have nominal diameters up to 29 mm and can be overdilated to 31 mm.^E3^

We believe that most patients with repaired TOF with transannular patch can have their own PVs reconstructed provided that the PV was not resected entirely during prior repair. Such repair would require less prosthetic material and, thus, may carry lower risk for endocarditis. It has been suggested that there is greater total burden related to foreign material; for example, a valve implanted within a degenerated conduit would convey greater risk than a valve implanted in native tissue, yet this opinion remains to be proved or refuted by any meaningful data.^E4^ Nonetheless, the most concerning postimplant complication of bovine jugular vein graft with either surgical or transcatheter implantation remains infective endocarditis.^6^ We reported an incidence of endocarditis in surgically implanted bovine jugular veins of 9.4%.^4^ After Melody valve implantation, the incidence of endocarditis can be as high as 3.1% per patient-year.^E4^ Risk factors for infective endocarditis appear to be residual gradients >15 mm Hg and younger age (younger than age 12 years).^E4^

We believe the reinforcement of the annulus and reconstruction of the leaflet prevents further dilation and will likely result in durable PV repair. Moreover, if the valve replacement is required at a later stage, it can be done percutaneously in the stabilized subpulmonary muscular conus.

## Conflict of Interest Statement

The authors reported no conflicts of interest.

The *Journal* policy requires editors and reviewers to disclose conflicts of interest and to decline handling or reviewing manuscripts for which they may have a conflict of interest. The editors and reviewers of this article have no conflicts of interest.

## Supplementary Data

Video 1 The senior author explains perioperative findings and procedures. Video available at: https://www.jtcvs.org/article/S2666-2507(25)00263-9/fulltext.
