# Supplementary figures and images for: Conus stabilization for pulmonary valve reconstruction after transannular patch
Source: JTCVS Tech. 2025 Jun 26;33:188–91. doi: 10.1016/j.xjtc.2025.06.014 (PMC12529722; doi:10.1016/j.xjtc.2025.06.014)

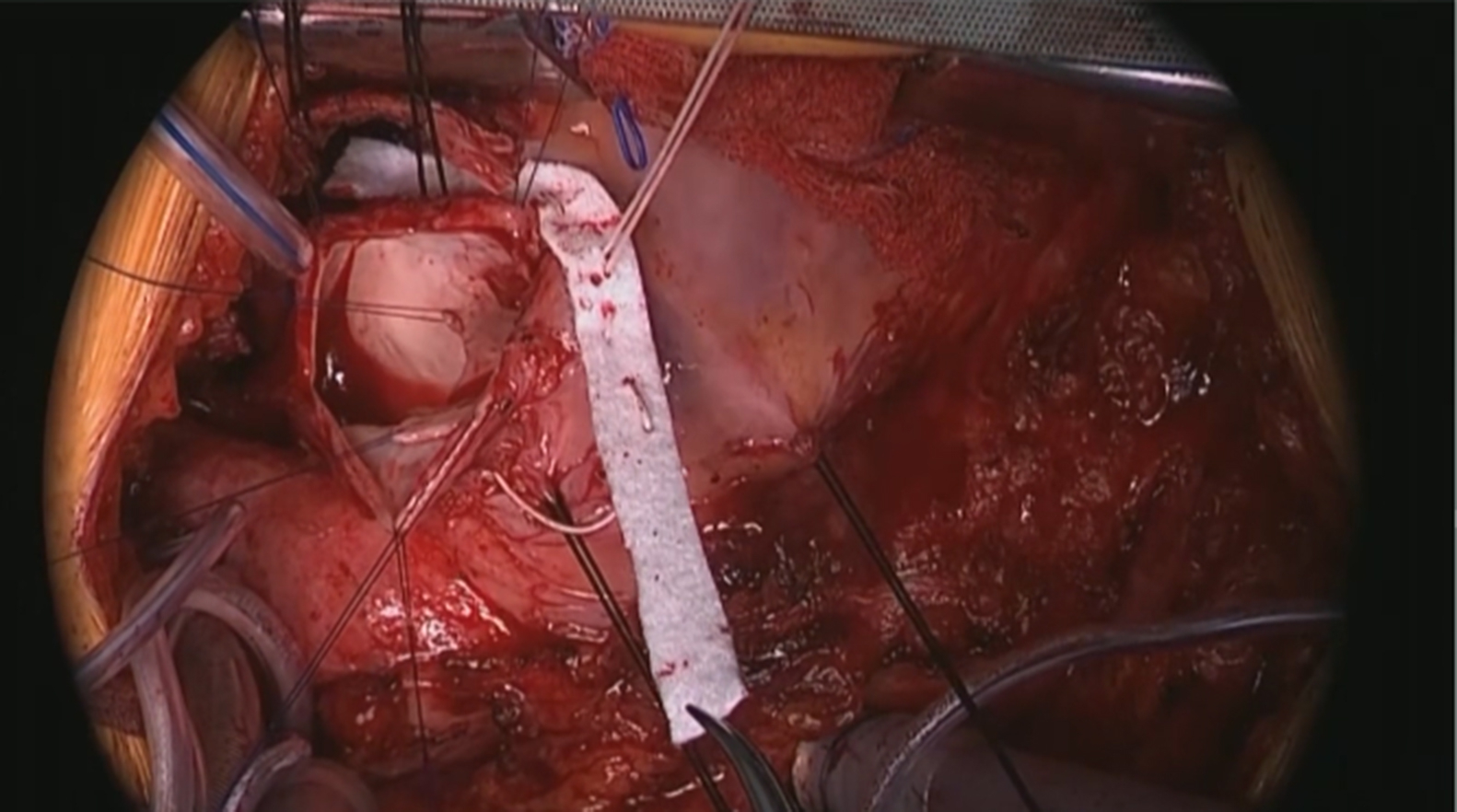

Supplement: Video 1 — The senior author explains perioperative findings and procedures. Video available at: https://www.jtcvs.org/article/S2666-2507(25)00263-9/fulltext. [file fx2.jpg]
